# Fast Track to Discover Novel Promoters in Rice

**DOI:** 10.3390/plants9010125

**Published:** 2020-01-18

**Authors:** Yo-Han Yoo, Yu-Jin Kim, Sunok Moon, Yun-Shil Gho, Woo-Jong Hong, Eui-Jung Kim, Xu Jiang, Ki-Hong Jung

**Affiliations:** Graduate School of Biotechnology & Crop Biotech Institute, Kyung Hee University, Yongin 17104, Korea; directorhan@khu.ac.kr (Y.-H.Y.); yujinkim@khu.ac.kr (Y.-J.K.); moonsun@khu.ac.kr (S.M.); koyoong@khu.ac.kr (Y.-S.G.); hwj0602@khu.ac.kr (W.-J.H.); alice804@khu.ac.kr (E.-J.K.); kangwuk97@khu.ac.kr (X.J.)

**Keywords:** rice, promoter trap, GUS, meta-expression analysis

## Abstract

Promoters are key components for the application of biotechnological techniques in crop plants. Reporter genes such as *GUS* or *GFP* have been used to test the activity of promoters for diverse applications. A huge number of T-DNAs carrying promoterless *GUS* near their right borders have been inserted into the rice genome, and 105,739 flanking sequence tags from rice lines with this T-DNA insertion have been identified, establishing potential promoter trap lines for 20,899 out of 55,986 genes in the rice genome. Anatomical meta-expression data and information on abiotic stress related to these promoter trap lines enable us to quickly identify new promoters associated with various expression patterns. In the present report, we introduce a strategy to identify new promoters in a very short period of time using a combination of meta-expression analysis and promoter trap lines.

## 1. Introduction

Promoter trap systems have been developed to identify the activity of endogenous promoters in transgenic plants carrying promoterless reporter systems near the right or left borders of their T-DNA via simple chemical assays. They are valuable tools to screen for specific reporter activity in a domain of interest. In general, β-glucuronidase (*GUS*) reporter genes are employed for this purpose. Insertion of T-DNA with multiple splice donor and acceptor sequences in front of a *GUS* gene within a transcriptional unit of the genome allows the generation of in situ gene fusions regardless of the insert position. Transcript expression can be visualized by the T-DNA insertion including the *GUS* reporter. To date, the expression patterns of more than 100 rice genes have been reported in PubMed Central by using this reporter system. In addition, expression pattern analyses have been used in functional studies of target genes [1,2,3,4,5,6].

The International Rice Functional Genomics Consortium has produced more than one million T-DNA/Ds/Tos17 insertion lines (Ds and Tos17 being two other types of insertion elements), and these insertions cover more than 60% of the total genes in the rice genome [7,8]. Of these, we produced 106,100 lines with T-DNA insertions and identified flanking sequencing tags for 59,707 T-DNA-insertion positions [9]. Because T-DNA is inserted randomly in both orientations, about half of these 59,707 positions are available for promoter traps. We previously reported that 10% (4/40) of the tested samples in a random screening showed *GUS* activity, indicating that the efficiency of the promoter trap system is 10% [10]. It is not easy to identify promoters with target expression patterns by random screening of thousands of promoter trap lines. On the other hand, expression analysis using the *GUS* reporter system has been used for the functional identification of a gene of interest.

With recent progress in transcriptome analysis technology, information regarding organ-specific genes or stress-specific expression patterns can be easily obtained from the huge amount of data available, and the development of promoters using this information is progressing [11,12]. Recently, we confirmed the expression of tandem duplicated genes that showed differential expression patterns in the embryos and endosperm of rice seeds through the production of promoter–*GUS* plants [13]. Using large-scale transcript information on various tissues or organs, it is possible to easily distinguish promoters driving organ- or tissue-specific expression patterns. However, for most crops, at least two generations are required to confirm the successful development of promoters using transformants.

In the present study, we performed an assay to identify novel promoters in as short a time as possible by integrating meta-expression data into a large-scale promoter trap system. To do this, we established a meta-expression database for various publicly available organ and tissue samples and then selected putative promoter trap lines for 100 genes showing root-, leaf-, pollen-, or seed-preferred expression patterns or ubiquitous expression. In a very short period (minimum two weeks to maximum three months), we identified a promoter trap line for each gene group showing preferred expression patterns in diverse tissues/organs or diverse abiotic stress responses. Here, we will introduce a fast trap method for the identification of interesting promoters in rice by using a combination of meta-expression data and global promoter trap lines.

## 2. Results and Discussion

### 2.1. Summary of the Promoter Trap Line Analysis Process

**Figure 1:**
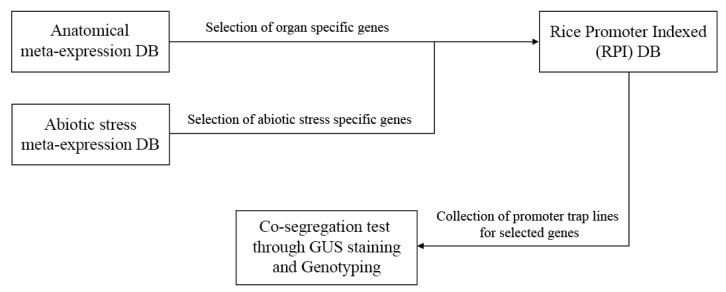
Workflow diagram summarizing the analysis process of the promoter trap line. DB: database.

In the present study, we explored a new approach to identifying novel genes using transcriptome data and promoter trap lines. Initially, we selected 700 organ-specific or abiotic stress-specific genes using an anatomical and stress meta-expression database. Then, a rice promoter-indexed (RPI) database was used to search for potential promoter trap lines for the selected genes. *GUS* expression was tested for several genes by GUS staining and genotyping. Finally, previous reports of gene expression analysis with the promoter trap system in the funRiceGenes database were examined. The analysis process for the promoter trap line is summarized in Figure 1.

The workflow illustrates the entire analysis process of the present study. First, we selected organ-specific or abiotic stress-specific genes from a meta-expression database using the k-means clustering (KMC) algorithm. Then, an RPI database was used to select potential promoter trap lines. Finally, co-segregation tests were performed by GUS staining and genotyping.

### 2.2. Integration of Annotated Rice Genes from the Rice Genome Annotation Project

Annotation data for rice genes were downloaded from the Michigan State University Rice Genome Annotation Project Database (RGAP), which provides sequence and annotation data for the rice genome [14]. We searched these data for annotated genes and identified 55,801 genes that were classified under LOC_id (e.g., LOC_Os07g40320).

### 2.3. Identification of Tissue/Organ-Preferred Genes in Rice Using Meta-Expression Data

**Figure 2:**
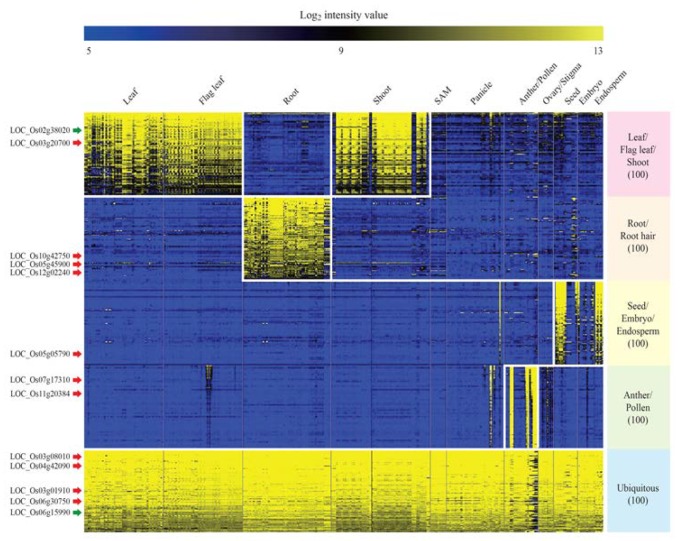
Heat map analysis of organ-specific genes and identification of five clusters.

To find organ-preferential genes among 55,801 rice genes, we used meta-anatomical expression profiles consisting of 983 rice Affymetrix array anatomical sample data points [15]. Next, clustering analysis was performed using a Euclidian distance algorithm, and genes were grouped into 20 anatomical clusters. Through this analysis, we found five anatomical clusters with organ-preferred expression patterns as follows: leaf/flag leaf/shoot, root, seed/embryo/endosperm, anther/pollen, and ubiquitous (Figure 2). We selected 100 genes in these five clusters. The anatomical meta-expression data consisting of 983 rice Affymetrix arrays for 500 genes are summarized in Table S1 [16].

We used KMC to separate genes into 20 clusters using a Euclidean distance matrix and selected five clusters based on the tissue-specific expression patterns of the genes (leaf/flag leaf/shoot, root, seed/embryo/endosperm, anther/pollen, and ubiquitous). In Figure 2, blue indicates the lowest expression level, and yellow indicates the highest expression level. Genes identified previously are indicated by red arrows, and genes newly discovered through the promoter trap system are indicated by green arrows. Detailed data on the anatomical expression analysis are presented in Table S1.

### 2.4. Validation of Promoters of Tissue/Organ-Preferential Genes Using the Promoter Trap System and Genotyping

We used an RPI database to secure potential promoter trap lines from selected genes. This database provides information on the location of T-DNA insertions and insertion vectors and the variety and orientation of promoterless *GUS*. Based on this, promoter trap lines could potentially be used to identify the activity of endogenous promoters from each gene cluster. Related information is listed in Table S2: leaf/flag leaf/shoot, 32 lines; root, 23 lines; seed/embryo/endosperm, 16 lines; anther/pollen, 23 lines; and ubiquitous, 64 lines [10].

**Figure 3:**
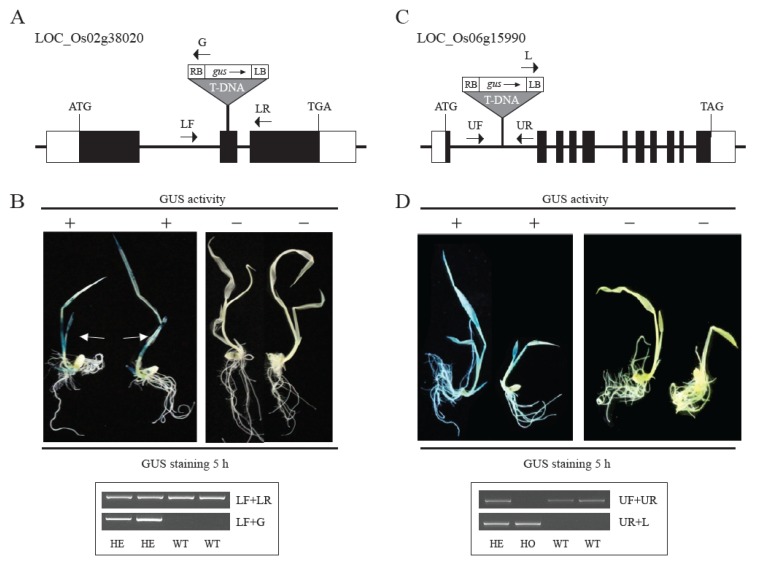
Confirmation of organ-specific genes using the promoter trap system. Schematic diagram of the T-DNA insertion site in *LOC_Os02g38020* of PFG 1C-011049 (**A**) and *LOC_Os06g15990* of PFG 3A-51959 (**C**). Black boxes represent exons; white boxes represent untranslated regions (UTRs); lines between boxes, represent introns; the gray triangle represents T-DNA insertion; small arrows, represent gene-specific primers for genotyping of the tagged gene. ATG and TGA indicate start and stop codons, respectively. GUS expression analysis and genotyping were performed for two PFG lines (**B**,**D**). GUS expression patterns were observed in leaves (indicated by arrows (**B**), and whole plants (**D**)). Genotyping identified wild-type, heterozygous, and homozygous progenies with T-DNA. WT, wild-type segregants of T-DNA insertional line; HO, homozygote; HE, heterozygote. The primer sequences are indicated in Table S5: LF, leaf forward primer, LR, leaf reverse primer; UF, ubiquitous forward primer; UR, ubiquitous reverse primer; L, Left border primer; G, GUS primer.

*LOC_Os02g38020* is preferentially expressed in the leaves, and a relevant promoter trap line with T-DNA inserted in the second exon was identified (PFG 1C-011049, Figure 3A). To observe the GUS staining pattern of this gene, we performed GUS staining of plants grown in Murashige and Skoog (MS) medium for a week. As expected, we observed a leaf-preferred GUS staining pattern for this gene. Genotyping analysis revealed that the stained plants were heterozygous, and the unstained plants were wild-type (Figure 3B), indicating that the GUS expression data represent the endogenous expression of the target gene. *LOC_Os06g15990* is expressed in all organs, and the T-DNA of PFG 3A-51959 is inserted in the first intron of the gene (Figure 3C). Using the same method mentioned above, GUS staining patterns were observed in the leaves and roots of plants (Figure 3D). Genotyping analysis also confirmed that all plants with *GUS* activity were heterozygous or homozygous. The two genes identified by GUS staining are indicated by green arrows in the heat map in Figure 2.

### 2.5. Abiotic Stress Analyses of Rice Genes via Meta-Expression Data

**Figure 4:**
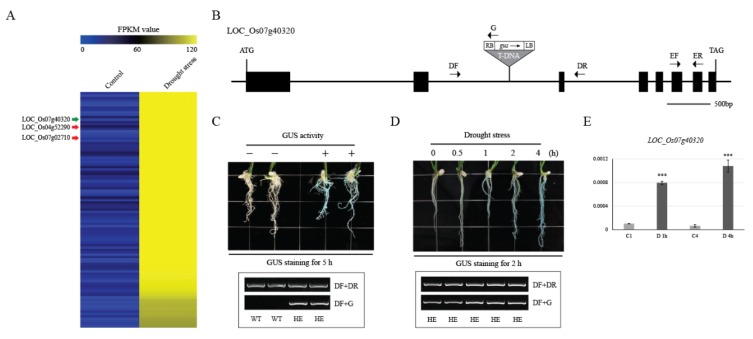
Heat map analysis of drought-inducible genes and confirmation using the promoter trap system. Using RNA-seq data processing, we identified 100 drought-inducible genes (**A**). A schematic diagram of the T-DNA insertion site in *LOC_Os07g40320* of PFG 3A-01968 is shown (**B**). To identify in planta expression of selected candidates, we germinated the seeds for one promoter trap line in Murashige and Skoog (MS) medium for 7 d and air-dried the plantlets for 0–4 h. Whole seedlings were then incubated in GUS-staining solution (**C**,**D**). Expression of *LOC_Os07g40320* was significantly up-regulated by drought stress, based on qRT-PCR (**E**). Blue indicates the lowest GUS expression level, and yellow indicates the highest GUS expression level. FPKM, fragments per kilobase per million fragments mapped (**A**). Black boxes, exons; white boxes, UTR; lines between boxes, introns; gray triangle, T-DNA insertion; small arrows, gene-specific primers (DF and DR, genotyping primer; EF and ER, expression primer). ATG and TGA indicate start and stop codons, respectively (**B**). C1, and C4, untreated control corresponding to plants under 1 h of drought stress (D 1 h) and 4 h of drought stress (D 4 h); *** *p* < 0.001 (**E**).

To identify drought-inducible genes, we used differentially expressed genes (DEGs) from an RNA-seq analysis reported previously [5]. Compared to the control, 100 genes with the highest fragments per kilobase per million fragments mapped (FPKM) values were screened during drought treatment (Figure 4A; Table S3) [17]. Next, we used a series of 13 expression datasets to find cold-inducible genes. These datasets include five types of abiotic stress: drought, salinity, cold, heat, and submergence. We identified cold-induced genes using KMC analysis under the Euclidian distance algorithm and selected the 100 genes with the highest average log2-fold-change values (treatment/control). (Figure S1; Table S4).

### 2.6. Evaluation of Promoter Trap Lines through a Literature Search

To evaluate the significance of our candidate genes for the trap promoter, we searched for relevant literature in the funRiceGenes database [18]. We identified reports that revealed *GUS* expression using a promoter trap line (Table 1) and we found that 15 genes showed organ-specific or abiotic stress-specific expression patterns. Among them, 11 genes showed GUS expression in various organs. They include *LOC_Os03g20700* (PFG 9-07117) in leaves; *LOC_Os05g45900* (PFG 3A-00457), *LOC_Os10g42750* (PFG 2B-60199), and *LOC_Os12g02240* (PFG 4A-50567) in root hairs; *LOC_Os05g05790* (PFG 1A-10540) in seeds; *LOC_Os11g20384* (PFG 1A-13819) and *LOC_Os07g17310* (PFG 2D-41188) in pollen; and *LOC_Os03g01910* (PFG 4A-04197), *LOC_Os03g08010* (PFG 5A-00191), *LOC_Os04g42090* (PFG 3A-05916), and *LOC_Os06g30750* (PFG 2D-00098) in all organs. Four of the genes were related to responses to various abiotic stresses, namely, *LOC_Os04g52290* (PFG 3A-03417) and *LOC_Os07g02710* (PFG 3A-13738) to drought and *LOC_Os01g31370* (PFG 3A-50649) and *LOC_Os03g49830* (PFG 1C-08613) to cold. The genes identified in previous reports are marked with red arrows in the heat map in Figure 2. These data further support the usefulness of our strategy to identify promoters of interest.

### 2.7. Validation of Drought-Inducible Genes Using the GUS Reporter System and qRT-PCR

We identified 65 potential promoter trap lines for 100 drought-inducible genes in the RPI database. Among these, the promoter trap lines of one gene (PFG 3A-01968 for *LOC_Os07g40320*) exhibited GUS expression in roots after plants were exposed to drought stress for 0, 0.5, 1, 2, and 4 h (Figure 4D) [5]. To ensure the accuracy of our GUS expression analysis, GUS staining was performed for the same time after stress treatment, and all plants used were heterozygotes. Interestingly, the longer the exposure to drought stress, the stronger the observed GUS expression. This drought-related expression was verified by qRT-PCR (Figure 4E). Our findings show that the promoter trap system is very effective in identifying the activity of promoters and could also enable researchers to develop novel promoters.

## 3. Materials and Methods

### 3.1. Integration of Whole Rice Genes from Public Data Source

We downloaded annotation data as an entire set from the RGAP web database (http://rice.plantbiology.msu.edu/pub/data/Eukaryotic_Projects/o_sativa/annotation_dbs/) [19]. From these data, we identified 55,801 genes annotated in rice chromosomes.

### 3.2. Collection of Transcriptome Data

Microarray datasets for meta-expression analysis were downloaded from the National Center for Biotechnology Information Gene Expression Omnibus (NCBI GEO) Affymetrix collections. To analyze anatomical expression profiles, we integrated anatomical data from the rice oligonucleotide array database ROAD [20]. To compile an abiotic stress database, we retrieved 14 expression dataset series, GSE92989, GSE38023, GSE37940, GSE33204, GSE31077, GSE28209, GSE26280, GSE25176, GSE24048, GSE23211, GSE21651, GSE18930, GSE16108, and GSE6901, from the NCBI GEO (https://www.ncbi.nlm.nih.gov/gds).

### 3.3. Classification of Organ-Preferential or Abiotic Stress-Responsive Gene Groups

To analyze anatomical data, we used the Affy package encoded in R language to normalize signal intensities and then transformed them to log_2_ values. The normalized data with Affymetrix anatomical meta-expression data were then used for KMC with Euclidean distance metric embedded in Multiple Experiment Viewer (MeV) software (version 4.9.0). Using this method, we identified 100 genes in each of five categories according to their expression patterns (leaf/flag leaf/shoot, root, seed/embryo/endosperm, anther/pollen, and ubiquitous). To compile abiotic transcriptome data, we clustered 100 genes that were preferentially expressed in drought or cold conditions using the same KMC algorithm. We selected only genes with an average log_2_-fold-change value (treatment/control) in a cluster greater than 1 (log_2_ value) and a *p*-value of less than 0.05 according to one-way analysis of variance (ANOVA) to ensure clear correlation. Finally, we used Illustrator software (Adobe Illustrator CS6) to produce heat map images [21].

### 3.4. Histochemical GUS Assay

T-DNA insertional lines generated from *Oryza sativa* ssp. Japonica of cv. Dongjin or Hwayoung were screened in this study. To examine GUS expression patterns, we germinated the seeds from three promoter trap lines in MS medium for 7 d in a controlled growth chamber at 28 °C/22 °C (day/night), with a 12 h photoperiod. Histochemical GUS staining was performed as described by Hong et al. [13]. The composition of the GUS staining solution was as follows: 100 mM sodium phosphate (pH 7.0), 5 mM potassium ferricyanide, 5 mM potassium ferrocyanide, 0.5% (*v*/*v*) Triton X-100, 10 mM EDTA (pH 8.0), 0.1% (*w*/*v*) 5-bromo-4-chloro-3-indolyl-*β*-d-GlcA/cyclohexylammonium salt, 2% (*w*/*v*) dimethyl sulfoxide, and 5% (*v*/*v*) methanol. Chlorophyll was removed in 70% ethanol [22]. To induce drought stress, plantlets were air-dried for 0.0, 0.5, 1.0, 2.0, or 4.0 h. Afterward, whole seedlings from all treatment groups were soaked in GUS staining solution before their roots were photographed with a camera (Canon EOS 550D; Canon, Tokyo, Japan).

### 3.5. Quantitative Real-Time PCR (qRT-PCR) Analysis

Our qRT-PCR analysis was conducted as follows. Roots were sampled from control and drought-treated plants and immediately frozen in liquid nitrogen. After total RNA was isolated with RNAiso (Takara Bio, Shiga, Japan), first-strand cDNA was synthesized using MMLV Reverse Transcriptase (Promega, WI, USA) and the oligo(dT) 15 primer. Synthesized cDNAs were amplified using SYBR Premix Ex Taq (TaKaRa), and qRT-PCR was performed on a Rotor-Gene Q instrument system (Qiagen, Hiden, Germany). To normalize the amplified transcripts, we used a primer pair for rice ubiquitin 5 (OsUbi5/Os01g22490) [23]. All the primers for these analyses are summarized in Table S5.

### 3.6. Analysis of Promoter Trap Lines via Literature Search

The funRiceGenes database was used to determine whether genes preferentially expressed in various organs and during abiotic stress had been studied using promoter trap lines (https://funricegenes.github.io/) [18]. In this database, information on 3148 functionally characterized genes is available. We parsed the functional roles for the 700 genes (five organ categories and two types of stress) using meta-expression data, which are summarized in Table 1.

## 4. Conclusions and Future Prospects

In the present study, we propose a fast trap method to find promoters of interest in the rice genome using a combination of transcriptome data and promoter trap lines. Although a number of promoters have been identified in plant species, most were part of functional genomics studies of genes of interest and some have not been evaluated by other supporting data such as genome-wide transcriptome data. For more accurate application, promoters that are more specifically suited to intended purposes are required. Our strategy will be useful in the identification of novel promoters based on expression patterns for diverse applications, including functional rice genomics and studies to modify interesting traits. In addition, the identification of interesting promoter elements could be adapted for the modification of vectors such as for efficient Cas9 expression.

## Figures and Tables

**Table 1:** Summary of promoter trap lines identified by GUS staining.

Category	Expression Pattern	Locus_ID	T-DNA Line No.	Putative Function	DOI References ^a^
Anatomy	Leaf/Flag leaf/Shoot	LOC_Os03g20700	9-07117	Magnesium chelatase	10.1093/pcp/pcg064
	Root (root hair)	LOC_Os05g45900	3A-00457	endonuclease/exonuclease/phosphatase family domain-containing protein	10.1186/s12284-018-0241-2
		LOC_Os10g42750	2B-60199	CSLD1, cellulose synthase-like family D	
		LOC_Os12g02240	4A-50567	expressed protein	
	Seed/Embryo/Endosperm	LOC_Os05g05790	1A-10540	double-stranded RNA binding motif-containing protein	10.1104/pp.014357
	Anther/Pollen	LOC_Os11g20384	1A-13819	SacI homology domain-containing protein	10.1186/s12284-018-0219-0
		LOC_Os07g17310	2D-41188	B12D protein	
	Ubiquitous	LOC_Os03g01910	4A-04197	transcription factor BTF3	10.1016/j.molp.2014.10.013
		LOC_Os03g08010	5A-00191	elongation factor Tu	
		LOC_Os04g42090	3A-05916	CPuORF7, conserved peptide uORF-containing transcript	
		LOC_Os06g30750	2D-00098	reticulon domain-containing protein	
Abiotic stress	Drought	LOC_Os04g52290	3A-03417	PPR repeat domain-containing protein	10.3389/fpls.2017.00580
		LOC_Os07g02710	3A-13738	expressed protein	
	Cold	LOC_Os01g31370	3A-50649	glycosyltransferase	10.3389/fpls.2017.01120
		LOC_Os03g49830	1C-08613	expressed protein	

^a^ Indicates digital object identifier (DOI). uORF; upstream open reading frame; PPR, pentatricopeptide repeat.

## Supplementary Materials

The following are available online at https://www.mdpi.com/2223-7747/9/1/125/s1, Table S1. Microarray data on genes strongly associated with leaves/flag leaves/shoots, roots/root hairs, seeds/embryos/endosperm, anthers/pollen, and all organs. Table S2. List of potential promoter trap lines selected from organ- or abiotic stress-specific expression patterns. Table S3. RNA-seq data of genes showing increased expression in response to drought stress. Table S4. Microarray data of genes showing increased expression in response to cold stress. Table S5. Primer sequences used for genotyping and qRT-PCR. Figure S1. Heat map analysis of cold stress-responsive genes. We used a KMC algorithm with a Euclidean distance matrix to cluster abiotic stress-responsive genes. To define stress responsiveness, we applied the following criteria: greater than an average of 1 log_2_-fold change (2-fold) for each stress and a *p*-value less than 0.05 according to one-way ANOVA. As a result, we identified 100 cold stress-responsive genes. Red indicates upregulation under stress vs. control; green indicates downregulation under stress vs. control. Genes identified previously are marked by red arrows. Detailed data on abiotic stress expression analysis are presented in Table S4.
